# Supplementary material for: Perceived Neighborhood Environment and Its Association with Health Screening and Exercise Participation amongst Low-Income Public Rental Flat Residents in Singapore
Source: Int J Environ Res Public Health. 2019 Apr 17;16(8):1384. doi: 10.3390/ijerph16081384 (PMC6517983; doi:10.3390/ijerph16081384)
Supplement: Supplementary file 1 [file ijerph-16-01384-s001.pdf]

International Journal of
Environmental Research and Public Health

MDPI

Supplementary Material

Perceived Neighborhood Environment and Its Association with Health Screening and Exercise Participation amongst Low-Income Public Rental Flat Residents in Singapore

Liang En Wee, Yun Ying Tammy Tsang, Sook Muay Tay, Andre Cheah, Mark Puhaindran, Jaime Yee, Shannon Lee, Kellynn Oen and Choon Huat Gerald Koh

Table S1: Principal components analysis of the modified 17-item Neighbourhood Environment Walkability Scale-Abbreviated (NEWS-A).

**Original subscales of the NEWS**	**Principal components derived from factor analysis**		
**Crime safety (7 items)**	Perceived safety and convenience (45.2% of variance)	Perceived physical environment (10.60% of variance)	Proximity to recreational areas (6.16% of variance)
Presence of litter/trash	0.345	**0.676**	0.153
Lighting/signage in neighbourhood streets	0.303	**0.616**	-0.034
Presence of pedestrians/people on the street	0.341	**0.637**	0.019
Observed interaction (eg. conversation) between pedestrians on the street	**0.678**	-0.099	-0.022
Perceived crime rate in neighbourhood	**0.728**	-0.112	0.158
Perceived safety when walking around neighbourhood by day	**0.818**	-0.134	0.097
Perceived safety when walking around neighbourhood by night	**0.730**	-0.108	0.096
**Land use access (2 items)**			
Physical barriers in neighbourhood (eg. uneven ground, steps)	0.298	**0.663**	-0.053
Easy to walk to bus & train stations	**0.795**	-0.043	0.004
**Land use diversity (8 items)**			
Grocery store (provision shop, wet market, supermarket)	**0.857**	-0.131	0.061

Eating place (hawker centre, coffee shop)	**0.729**	-0.063	-0.178
Financial services (bank/ATM/post office)	**0.761**	0.031	-0.069
Religious institution (eg. temples, churches)	**0.706**	-0.071	-0.029
Dispensaries (pharmacies, medicinal shops)	**0.845**	-0.128	0.065
Medical services (clinics/dentals)	**0.863**	-0.111	0.038
Community activity centre (eg. community club, senior activity centre)	**0.787**	-0.097	-0.188
Recreational areas (eg. park, swimming pools, gym or fitness facility)	-0.080	-0.030	**0.946**

© 2019 by the authors. Submitted for possible open access publication under the terms and conditions of the Creative Commons Attribution (CC BY) license (http://creativecommons.org/licenses/by/4.0/).
